# Nuclear MEK1 Sequesters PPARγ and Bisects MEK1/ERK Signaling: A Non-Canonical Pathway of Retinoic Acid Inhibition of Adipocyte Differentiation

**DOI:** 10.1371/journal.pone.0100862

**Published:** 2014-06-24

**Authors:** Sandeep Dave, Ravikanth Nanduri, Hedwin Kitdorlang Dkhar, Ella Bhagyaraj, Alka Rao, Pawan Gupta

**Affiliations:** CSIR-Institute of Microbial Technology, Chandigarh, India; Aligarh Muslim University, India

## Abstract

Uncontrolled adipogenesis and adipocyte proliferation have been connected to human comorbidities. Retinoic acid (RA) is known to inhibit adipocyte differentiation, however the underlying mechanisms have not been adequately understood. This study reports that RA acting as a ligand to RA receptors (RARs and RXRs) is not a *sine qua non* to the inhibition of adipogenesis. Our intriguing observation of a negative correlation between increased retinoylation and adipogenesis led us to explore retinoylated proteins in adipocytes. Exportin (CRM1) was found to be retinoylated, which in turn can affect the spatio-temporal regulation of the important signaling molecule mitogen-activated protein kinase kinase 1 (MEK1), likely by disrupting its export from the nucleus. Nuclear enrichment of MEK1 physically sequesters peroxisome proliferator-activated receptor gamma (PPARγ), the master regulator of adipogenesis, from its target genes and thus inhibits adipogenesis while also disrupting the MEK1-extracellular-signal regulated kinase (ERK) signaling cascade. This study is first to report the inhibition of adipocyte differentiation by retinoylation.

## Introduction

All-trans-retinoic acid (atRA) is an active hormonal form of vitamin A. RA serves as a ligand and regulates gene expression through two classes of nuclear retinoid receptors, RA receptors (RARα, RARβ, RARγ) and retinoid X receptors (RXRα, RXRβ, RXRγ) [1],[2]. It is generally accepted that nuclear retinoid receptors mediate the biological activity of RA. However, a correlation is not always seen between RA's affinity to RAR and its biological potency [3]–[9]. Non-genomic mechanisms such as retinoylation (acylation by RA) of proteins have been identified but not adequately addressed [6],[10]–[16]. Positive correlations have been reported between the retinoylation reaction and testosterone biosynthesis [17], embryonic carcinoma cell differentiation [18],[19], and fibroblast cell growth [20],[21].

RA incorporation with proteins have been validated in both *in vitro* [10],[22],[23] and *in vivo* experimental setups [10],[23]–[25]. Significant retinoylated protein mass has been observed in tissues or cells of testis, brain, kidney, liver and fibroblasts, yet only a short list of retinoylated proteins have been identified. The list includes regulatory subunits of cAMP-dependent protein kinase I and II, vimentin, the cytokeratins, ribonucleotide reductase, 2-oxoglutarate/malate carrier protein, and nuclear proteins such as histone deacetylase 3 (HDAC3) [26]–[31]. The covalent linkage between RA and proteins is probably a thioester and involves formation of a coenzyme A (CoA) intermediate [13],[23],[32],[33]. The retinoylation reaction displays rapid kinetics (12–24 h). The optimal concentration of RA for the retinoylation reaction is 100 nM–1 µM [22],[34],[35]. atRA is an inhibitor of adipocyte differentiation [7],[36] and an effective anti-obesity nutritional supplement [37], however the mechanism for these actions has remained elusive. This inhibitory effect depends on the concentration and stage of differentiation of the adipocytes. atRA has been clearly shown to inhibit adipocyte differentiation at relatively high doses (100 nM–10 µM) and in a narrow window (0–48 h) at the early stages of adipogenesis [36].

Retinoylated proteins have been identified in 3T3 fibroblasts, an upstream lineage of adipocytes [8], a finding which supports the hypothesis that retinoylation of the adipocyte proteome may modulate adipogenesis. The mitogen-activated protein kinase kinase 1 (MEK1)/extracellular-signal regulated kinase (ERK) signaling pathway is known to promote adipogenesis [38],[39]. MEK1 has a nuclear export sequence (NES) in its N-terminal region and its nucleo-cytoplamic export can be directly regulated by exportin (CRM1) [40],[41]. MEK1 is also known to directly interact with the master regulator of adipogenesis, peroxisome proliferator-activated receptor gamma (PPARγ) [42]. We were therefore set to (i) investigate retinoylated proteins in 3T3-L1 adipocytes (ii) determine how CRM1 retinoylation affects (MEK1)/(ERK) signaling and (iii) investigate the transcriptional activity of PPARγ, the master regulator of adipogenesis.

Here in this study, biochemical experiments and site directed mutagenesis revealed that CRM1 is retinoylated. Retinoylation of CRM1 disrupts MEK1 nuclear egress and bisects the MEK1- ERK signaling cascade. Further, nuclear MEK1 sequesters the master regulator PPARγ, thereby disrupting its transcriptional activity. This is the first report of retinoylation of a protein that partially explains atRA mediated inhibition of adipogenesis.

## Materials And Methods

### Cell Culture And Differentiation

3T3-L1 mouse embryonic fibroblasts were procured from the cell repository at the National Centre for Cell Science, Pune, India and were cultured as described elsewhere [43]. Briefly, cells were cultured in growth medium containing 10% bovine calf serum (HyClone) in Dulbecco's modified Eagle's medium (DMEM) (delipidated). For the differentiation of preadipocytes, two days after the confluence, the cells were stimulated with differentiation medium (DM) in DMEM containing 10% dextran-coated charcoal*-*treated fetal bovine serum (DCC-FBS), 167 nmol/l insulin, 0.5 µmol/l isobutylmethylxanthine, and 1 µmol/l dexamethasone for 2 days. On day 2, the DM was replaced with post-differentiation medium (PDM) containing 10% DCC-FBS and 167 nmol/l insulin. PDM was repleted every three days. [3H]atRA (100 nM–1 µM) was added to the DM for 48 h along with CoA (10 µM–100 µM) in the experimental and control cells. Preadipocyte and adipocyte were treated with vehicle or test compounds in relevant media for time points as mentioned in the figure legend.

### *In Vitro* Retinoylation

atRA was converted to retinoyl CoA as described previously [10],[23]. Extent of retinoylation is dependent on CoA and all the reactions were performed in the presence of CoA [23],[44]. Briefly, a reaction mixture of 280 nM atRA or [^3^H]atRA, 10 mM ATP, 0.15 mM CoA, 27 mM MgCl_2_, 1 mM DTT, 50 mM sucrose, and 0.1 M Tris-HCl buffer (pH 7.4) in a final volume of 0.1 ml was incubated with protein/peptide (100 µg of protein) for the *in vitro* retinoylation reaction. The mixture was incubated at 37°C for 15 min and then extracted using the Bligh-Dyer method [45]. This extraction was repeated six times or until <300 cpm/ml was present in the supernatant fraction to rule out the presence of any unbound atRA. The delipidated pellet was dried and dissolved and radioactivity was measured by liquid scintillation spectroscopy using a Wallac-1450 Microbeta Trilux (Perkin Elmer, Waltham, MA).

### *In Vivo* Retinoylation And Incorporation Of Covalently Bound Radiolabelled Retinoids

Cell medium was enriched with appropriate amounts of atRA without compromising the viability of the cells [29]. [^3^H]atRA was dissolved in ethanol and the solution containing 100 nM [^3^H]atRA was added to cells along with CoA in DM [13]. The radioactivity in the delipidated residue was measured by liquid scintillation. Delipidized cell homogenates were dissolved in 1% SDS and digested with 0.4 mg proteinase K at 37°C for 1 h, as described elsewhere [8],[16]. Further BSA (50–100 µg/ml) was added followed by TCA precipitation. After centrifugation at 13000× g for 10 min, the radioactivity in both the supernatant solution and the precipitate was determined. Alkaline methanolysis was performed and processed as described previously [13],[25],[32].

### Immunoprecipitation And Western Blot Analysis

atRA-conjugated endogenous complexes in 3T3-L1 adipocytes were precipitated with anti-RA (AbD Serotec, UK or raised in house). These antibodies have specificity only for RA-conjugated regions of proteins, thus, free RA or proteins do not bind. Endogenous expression of proteins was monitored by western blot analysis of cell lysates and constituted the input. Antibodies specific for ERK, phospho-ERK (Cell Signaling and Santa Cruz), FLAG (Sigma), βactin, RARα and γ, PPARγ, pPPARγ, CEBPα, CEBPβ, CRM1, MEK1, HDAC3, vimentin (Santa Cruz) were used for the study, with appropriate secondary antibodies.

### Antibody Generation

Antibodies were generated in rabbit after conjugation of atRA to inert carrier matrix BSA/KLH as described previously [46].

### Microscopy

Immunofluorescent staining was performed as described previously [47]. Briefly, the cells were fixed with 4% PFA and permeabilized with 0.2% Triton X-100 for 5 min. Thereafter, the cells were incubated with antibody (1∶50 for endogenous or 1∶100 for overexpressed CRM1 and MEK1) for 45 min followed by incubation for 45 min with fluorescently conjugated secondary antibodies (as described in figure legends). Localization and fluorescence was observed under an LSM 510 Meta Carl Zeiss confocal microscope and a Nikon A1R confocal microscope.

### Preparation Of Cell Lysates And Isolation Of Nuclear And Cytoplasmic Fractions

Cells were collected and cell lysates prepared for isolation of nuclear and cytoplasmic fractions as described previously [48].

### Oil Red O Staining

Cells were washed with PBS and fixed for 10 min with 4% paraformaldehyde in PBS (pH 7.4). Cells were then stained for 30 min with Oil Red O (0.5 g in 100 ml isopropanol) as described earlier [49]. In some wells, Oil Red O dye retained in the cell was quantified by elution into isopropanol, and OD_500_ was measured.

### Sirna-Mediated Knockdown In Cultured Cells

siRNAs were purchased from Santa Cruz Biotechnologies and Qiagen [MEK1: Mm_Map2k1_3 FlexiTube siRNA (NM_008927)- SI01299613 and Mm_Map2k1_7 FlexiTube siRNA (NM_008927)- SI02710120]. siRNAs were transfected into 3T3-L1 cells at 50 nM final concentration using Lipofectamine (Invitrogen) according to the manufacturer's instructions. Cells were assayed 48–72 h after siRNA transfections.

### Rna Isolation And Quantitative Rt-Pcr

As described previously [47]. Briefly, cells were harvested in 1 ml of Trizol reagent (Invitrogen) and RNA was extracted according to the manufacturer's instructions. cDNA synthesis was performed with the RevertAid First Strand cDNA Synthesis kit (Fermentas) for RT-PCR and quantitative real-time RT-PCR (qPCR). qPCR was performed in 96-multiwell plates with a quantitative real-time PCR kit (Invitrogen). PCR was performed in an iCycler iQ real-time detection system (Bio-Rad), and the PCR baseline-subtracted data were computer generated as described by the manufacturer (Bio-Rad). 18S rRNA and β-actin were used as reference housekeeping genes for normalization.

### Chromatin Immuno-Precipitation (chip) Assay

ChIP assays were performed as described previously [50]. Briefly, the DNA and proteins were cross-linked by formaldehyde in 3T3-L1 cells from different experimental sets. Proteins were then immunoprecipitated using the indicated antibodies or rabbit IgG as mock control. qPCR amplification was performed using promoter-specific oligonucleotide primers from DNA extracted of the immunoprecipitates to amplify the PPARγ response element (PPRE) sequence stretch in the mouse aP2 promoter (5′GAGCCATGCGGATTCTTG3′ and 5′CCAGGAGCGGCTTGATTGTTA3′), and in the mouse Lpl promoter (5′CCTCCCGGTAGGCAAACTGGA3′ and 5′CCACTGCACAGCTGTTTAAGTGACTGG3′).

### Analysis Of Sequence Contexts Around Cysteines Of Known Retinyolated Proteins

Protein sequences for seven experimentally known retinyolated proteins were retrieved from Swiss-Prot. From these sequences, fixed size windows of residues lengths 21 at and around cysteines were extracted using the online tool Glyseq Extractor [51]. A total of 52 such windows were obtained representing all cysteines present in retinyolated proteins. Sequence weblogos were developed for these sequences using online tools available at www.weblogo.berkeley.edu/ and www.twosamplelogo.org/.

### In-Gel Digestion And Mass Spectrometry

For peptide mass fingerprinting (PMF), gel bands of interest were treated for trypsin (Sigma) in-gel digestion and the mass lists were generated by MALDI-TOF mass spectrometry on an ABI voyager DE STR mass spectrometer (Applied Biosystems) with the tryptic peptides using cyano-4-hydroxycinnamic acid as a matrix in a positive ion reflection mode. Data Explorer software was used for converting the raw data into a peak list. For protein identification the peak list generated was used to query all mouse NCBI nr sequence database entries using the Mascot software package (Matrix Sciences, London, U.K.). The PMF search was performed with a peptide mass tolerance of 100 ppm. The number of allowed missed cleavages was one. Peaks corresponding to contamination, e.g. trypsin autolysis peaks, matrix cluster ions and masses of tryptic peptides from keratin were excluded. Identified proteins were listed in Table 1.

**Table 1:** Included in this list are the proteins known and characterized as being retinoylated.

Protein Name	Gene ID	Protein ID
Oxoglutarate/malate carrier protein (Cys184)	NM_003562.4	NP_003553.2
cAMP-dependent protein kinase type I-alpha regulatory subunit	NM_002734.3	NP_002725.1
cAMP-dependent protein kinase type II-alpha regulatory subunit	NM_004157.2	NP_004148.1
Cytokeratinsin	NM_008470.1.	NP_032496.1
HDAC3	NM_010411.2	NP_034541.2
CRM1	NM_001035226.1	NP_001030303
Vimentin	NM_011701.4	NP_035831.2

### Plasmids

pFLAG-CRM1 was procured from Addgene (plasmid 17647) submitted by Dr. X. W. Wang. Cys 528, 585 to tryptophan substitutions in CRM1 were introduced via site-directed mutagenesis (Quickchange kit, Stratagene). The GFP-tagged MEK1 expression plasmid was a kind gift from Dr. Rony Seger (Weizmann Institute of Science, Rehovot, Israel). ΔN-EE-MEK1 (Export deficient mutant, constitutively active lacking NES) was prepared as described previously [42],[52].

### Reagents

Insulin, atRA, dexamethasone, 3-isobutyl-1-methylxanthine, Oil Red O, MG132, cycloheximide (CHX), aldehyde dehydrogenase (ALDH) inhibitor diethylamino-benzaldehyde (DEAB) and CoA were purchased from Sigma. DMEM, Trizol, fetal bovine serum, and penicillin–streptomycin were purchased from Invitrogen. Fetal calf serum was purchased from Hyclone. [^3^H]atRA, 50 Ci/mmole was purchased from Perkin Elmer. All chemicals used were of analytical grade.

### Statistics

Results are expressed as the mean ± SD unless otherwise noted. SigmaPlot (SyStat Software) and/or SPSS (IBM) were used for statistical analysis. Two-tailed Student's t-tests were performed to obtain P values. Statistical significance was established at * P<0.05. The efficiency of PCR amplification for each gene was calculated by the standard curve method (E = 10^−(1/log slope)^).

## Results

### Effect Of Atra Concentration On Protein Retinoylation And Adipogenesis In 3t3-L1 Cells

**Figure 1:**
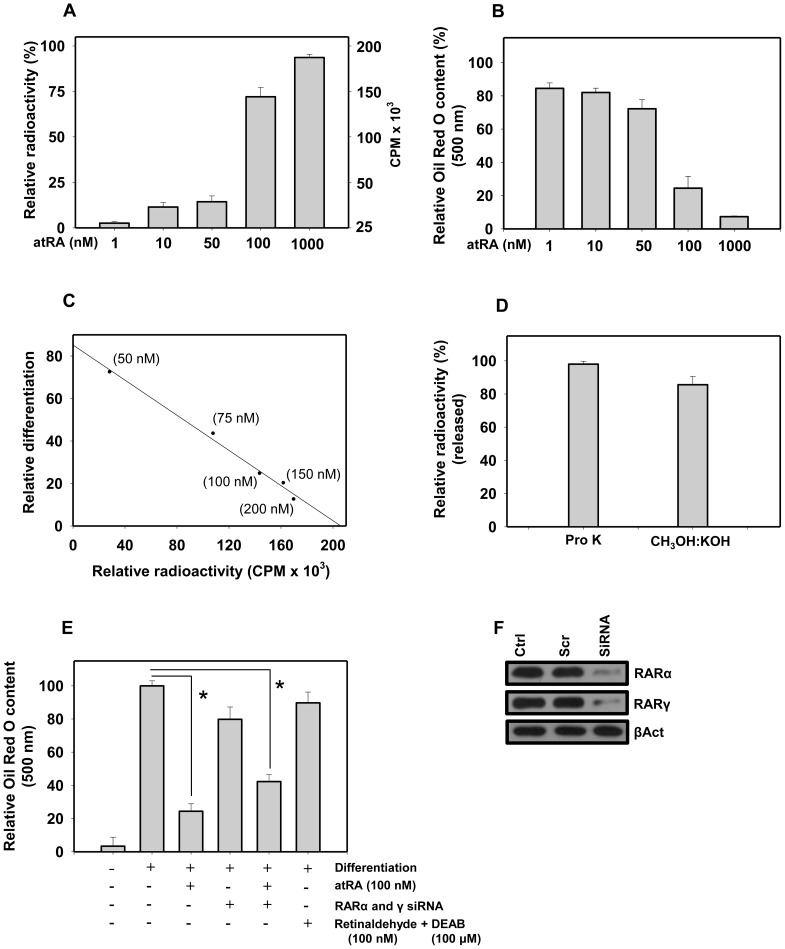
Retinoylation of proteins in 3T3-L1 adipocytes and inhibition of adipocyte differentiation. (**A**) Covalent conjugation of atRA in 3T3-L1 adipocyte proteome treated with log concentration series of [^3^H]atRA (1 nM–1 µM) for a period of 48 h along with the DM containing CoA. (**B**) After the above mentioned treatment the cells were kept for an additional 24 h in regular medium and extent of differentiation was monitored by Oil Red O staining which was spectrophotometrically determined at 500 nm. (**C**) The extent of retinoylation and adipocyte differentiation showed negative correlation with Pearson coefficient, r = −0.99553. (**D**) Release of retinoylated compounds from delipidated media after treatment with proteinase K or alkaline methanolysis. (**E**) Inhibition of adipocyte differentiation by atRA (100 nM) in RARα and γ knockdown background, or by retinaldehyde (100 nM) treatment along with ALDH inhibitor DEAB (100 µM). (**F**) Immunoblot for RARα and γ genes silencing. All experiments were repeated at least three times. Error bars show SD. *P<0.05 vs. controls.

Confluent 3T3-L1 preadipocytes were incubated with a log concentration series of [^3^H]atRA (1 nM–1 µM) for a period of 48 h along with DM, which was later switched to regular medium for another 24 h. A significant linear increase in the retinoylated protein was observed in the delipidated cell extract at and around 100 nM atRA, whereas a smaller increase was observed at 1 and 10 nM (Fig. 1A). However, an opposite effect on adipocyte differentiation was observed with increasing atRA concentrations, as monitored by Oil Red O staining. There was no significant modulation of adipocyte differentiation evident at 1 and 10 nM, however almost 80% inhibition was seen at 100 nM and the extent of differentiation was negligible at 1000 nM (Fig. 1B). Further, the extent of retinoylation negatively correlated with the extent of differentiation (Fig. 1C). We then subjected the delipidated cells to proteinase K and alkaline methanolysis (CH_3_OH:KOH). With proteinase K, 97% radioactivity was converted into a soluble form, while 85% of the radioactivity was released with CH_3_OH:KOH (Fig. 1D). Mass spectrometry identified methyl retinoate as the major product of this mild hydrolysis. These results have earlier indicated retinoylation of protein that involves covalent conjugation and probably formation of a thioester bond [13],[25],[32]. It has been shown that atRA is effective during the first 24 to 48 h in inhibiting adipocyte differentiation, which is so far understood to be redundantly mediated by RARα and γ [53],[54] in a way that does not involve direct binding to the target gene [7],[55],[56]. atRA seems to inhibit a step later to CCAAT/enhancer binding protein (CEBPβ) induction, however, the underlying molecular mechanism is not known. We were surprised to find that atRA could prevent adipocyte differentiation by classical and nonclassical pathways as well (Fig. 1E).

RARα and γ silenced background (via siRNA) (Fig. 1F) only partially rescued atRA inhibition of adipocyte differentiation, suggesting that there is an alternative mechanism which does not involve the RARs. Further, retinaldehyde plus ALDH inhibitor DEAB treatment was not effective in inhibiting adipocyte differentiation (Fig. 1E), which suggests an indispensable role of the carboxyl group of atRA in inhibition of adipocyte differentiation. These data suggest that the inhibitory effects of atRA on adipocyte differentiation involve not only ligand bound RAR, but are also governed by nonclassical pathways.

### Identification Of Retinoylated Proteins Separated By Sds-Page

**Figure 2:**
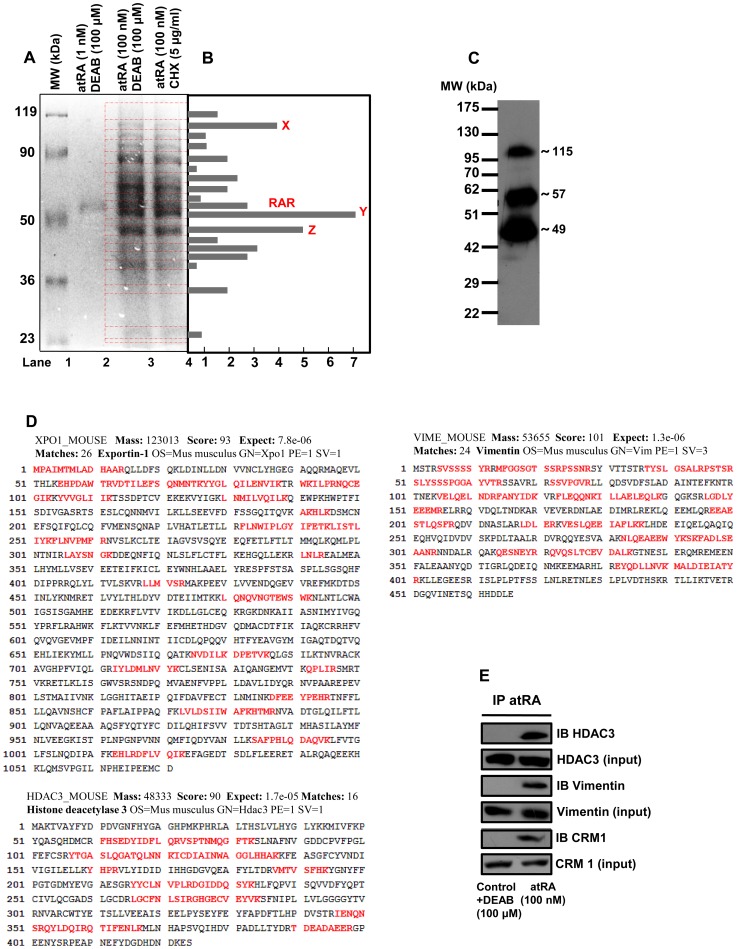
Separation and identification of retinoylated proteins. (**A**) Forty eight hours after [^3^H]atRA (100 nM) treatment along with DM containing CoA or other treatments, cells were delipidated and proteins that were covalently conjugated to atRA were pulled down by antibody specific for bound RA conjugated to any protein. Pulled down proteins were separated by SDS-PAGE (without β-mercaptoethanol) and subjected to Coomassie staining for subsequent mass spectrometry analysis. (**B**) Gel slices were excised and radioactivity incorporated in individual slices was monitored by liquid scintillation and normalized relative to radioactivity in five random gel slices with no protein band. (**C**) Bands specifically corresponding to retinoylated proteins were identified by immunoblot by antibody specific for bound RA followed after pull down using the same antibody. (**D**) Mass spectrometric identification and peptide coverage of retinoylated proteins (amino acids represented in red color are with the maximum identity). Gel slices corresponding to protein bands were excised. Proteins were digested in gel, followed by the extraction of tryptic peptides which were analyzed by peptide mass fingerprinting on MALDI-TOF (ABI voyager DE STR mass spectrometer). (**E**) Immunoprecipitation by antibody specific for bound RA followed by immunoblot using antibody to specific proteins identified validates protein specific retinoylation. All experiments were repeated at least three times.

Confluent 3T3-L1 cells were incubated with 100 nM [^3^H]atRA under standard conditions as described above. Effects of endogenous RA were ruled out by performing the experiments in delipidiated media and in the presence of ALDH inhibitor. *De novo* translation was blocked by the protein synthesis inhibitor CHX. Delipidated cell extracts were immunoprecipitated with antibodies specific for conjugated RA. Precipitated proteins were separated by SDS-PAGE (without β-mercaptoethanol) and stained (Fig. 2A). The proteins in the gel were transferred to membranes which were sliced and monitored for radioactivity (Fig. 2B). In the presence of 1 nM [^3^H]atRA along with 100 µM DEAB, a single band was observed around 50–58 kDa, which corresponds to RARα and γ (Lane 2, Fig. 2A). At 100 nM [^3^H]atRA, the pattern of bands observed was quite similar to that observed after treatment with 100 nM [^3^H]atRA in the presence of CHX (5 µg/ml) (Lane 3,4 Fig. 2A). These bands could be retinoylated proteins or their binding partners. The bands slices which do not have much of radioactivity associated could very well be the binding partners and not retinoylated proteins as such. This was clarified by liquid scintillation monitoring of incorporated radioactivity in individual slices. Bands x, y and z corresponding to molecular weights ∼115, 57, and 49 kDa respectively, incorporated significant radioactivity and seemed to be retinoylated (Fig. 2B). This was confirmed by immunoprecipitation followed by western blot using the same antibody (Fig. 2C). The protein bands were tryptic-digested and subjected to mass spectrometry analysis. CRM1, vimentin and HDAC3 were among the identified proteins, corresponding to bands (x) 115, (y) 57 and (z) 49 (Fig. 2D). Immunoblotting with protein-specific antibodies after immunoprecipitation with antibodies specific for protein conjugated RA, further validated these results (Fig. 2E). This study to the best of our knowledge is first to report retinoylation of CRM1 and therefore we pursued further consequent modulation of associated adipogenesis events.

### Crm1 Retinoylation Bisects The Mek1/erk Signaling Cascade By Nuclear Sequestration Of Mek1

**Figure 3:**
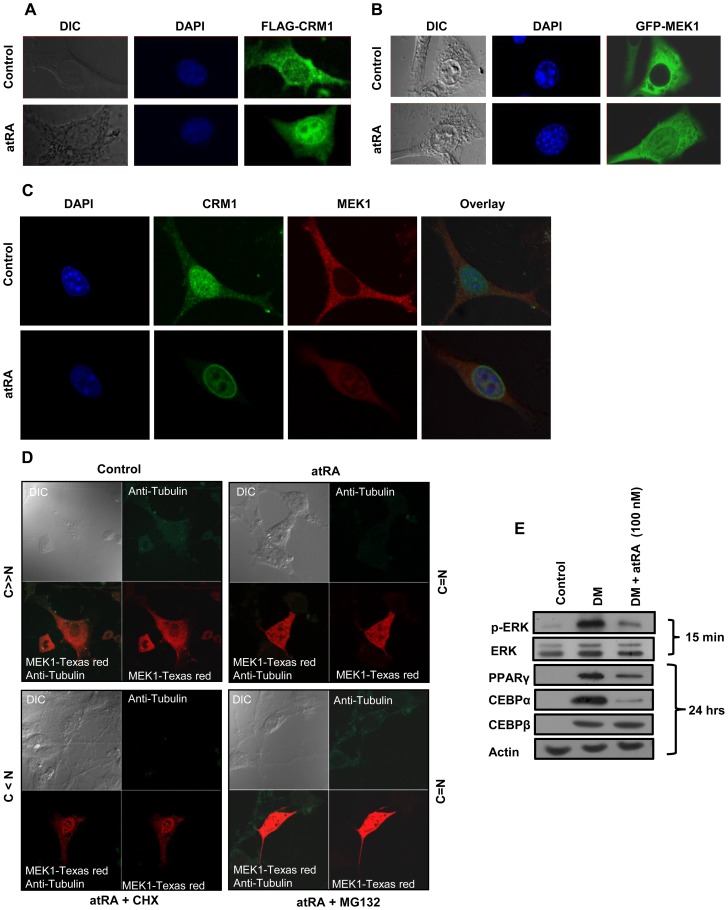
atRA modulation of nucleocytoplasmic distribution of CRM1 and MEK1. 3T3-L1 cells treated with DM containing CoA along with atRA (100 nM) or vehicle control for 48 h prior to fixation were transfected with FLAG-hCRM1 or GFP-hMEK1 expression constructs 8 h prior to fixation. Consequently, cells fixed and subjected to immunofluorescent staining reveal; (**A**) Immunofluorescence images of exclusive nuclear localization (C<<N) of CRM1 using anti FLAG Ab (secondary antibody conjugated to FITC) and, (**B**) nucleocytoplasmic localization (C = N) of MEK1 (GFP) in cells treated with atRA. (**C**) Representative projection images showing localization of endogenous MEK1 and CRM1, visualized by confocal microscope (Nikon A1R) anti MEK1 (secondary antibody conjugated to Texas Red) and anti CRM1 antibody (secondary antibody conjugated to FITC). DAPI stain was used for nuclear staining. (**D**) Endogenous MEK1 localization in presence of atRA along with cycloheximide (5 µg/ml) and MG132 (10 µM). The bars next to the images represent the scores of subcellular distribution (C<<N, exclusive nuclear; C = N, both nuclear and cytosolic; C>>N, exclusive cytosolic and C<N nuclear localization) among morphologically viable 300 cells. In atRA treated cells, nuclear export of MEK1 by CRM1 is abrogated. (**E**) Expression or activation/modification of endogenous ERK, CEBPα,β, PPARγ to DM and atRA treatment as monitored at mentioned timepoints. atRA treatment abrogated DM triggered ERK phosphorylation/activation (15 min) and PPARγ and CEBPα (24 h) expression. All experiments were repeated at least three times. Error bars show SD. * P<0.05 vs. controls.

One important role of the nuclear export protein CRM1 is the maintenance of cytosolic localization of certain regulatory molecules [40],[57]–[59]. We first investigated the localization of ectopically expressed hCRM1 in control and atRA stimulated cells (Fig. 3A). Ectopically-expressed hCRM1 was associated with the nuclear envelope/rim and was nucleocytoplasmic (C = N), however atRA treatment resulted in its exclusive localization to the nuclear envelope (C<<N). The MEK1/ERK signaling pathway is known to promote adipogenesis by enhancing PPARγ and CEBPα gene expression [38],[39], a step later to CEBPβ induction and a cascade which is inversely regulated by atRA [7]. The cytoplasm and nuclear shuttling of MEK1 is regulated by binding of CRM1 to its NES [40],[57],[58]. Interestingly, atRA treatment, much like Leptomycin B (LMB) [60],[61], led to nucleocytoplasmic localization (C = N) of ectopically expressed GFP-hMEK1, unlike in control cells where it was exclusively cytoplasmic (C>>N) (Fig. 3B). We next aimed to determine co-localization of endogenous CRM1 and MEK1 to these treatments. Expectedly, atRA treatment likewise resulted in CRM1 being majorly localized to the nuclear envelope/rim (C<<N), while MEK1 was nucleocytoplasmic (C = N) with no colocalization, which is suggestive of atRA-mediated abrogation of MEK1 export by CRM1 (Fig. 3C), possibly because of retinoylation of CRM1. No significant colocalization was observed in control cells, which could be due to the more rapid kinetics of MEK1 export [62]. To minimize the translational noise in our atRA treatment experiments, *de novo* MEK1 synthesis was blocked by the protein synthesis inhibitor CHX, and mostly nuclear localization (C<N) was observed (Fig. 3D). Further, to rule out basal degradation contributing to the atRA induced phenomenon, pretreatment with MG132 was performed (Fig. 3D). The results indicated that basal degradation was in no way contributory to the observed phenomenon. We then investigated whether the sequestration of MEK1 in the nucleus has any bearing on the MEK1/ERK signaling cascade (at 15 min) and, further, on the downstream target genes PPARγ and CEBPα (at 24 h) (Fig. 3E, Fig. S1A). Interestingly, unlike the control (with DM), atRA treatment (along with DM) abrogated ERK phosphorylation/activation and consequently, PPARγ and CEBPα expression. These results illustrate that at least one mechanism of adipogenesis inhibition by atRA involves selective nuclear accumulation of presynthesized MEK1 without effects on the localization of certain key effector downstream kinases.

### Nuclear Mek1 Sequesters Pparγ And Restricts Its Genomic Functions

**Figure 4:**
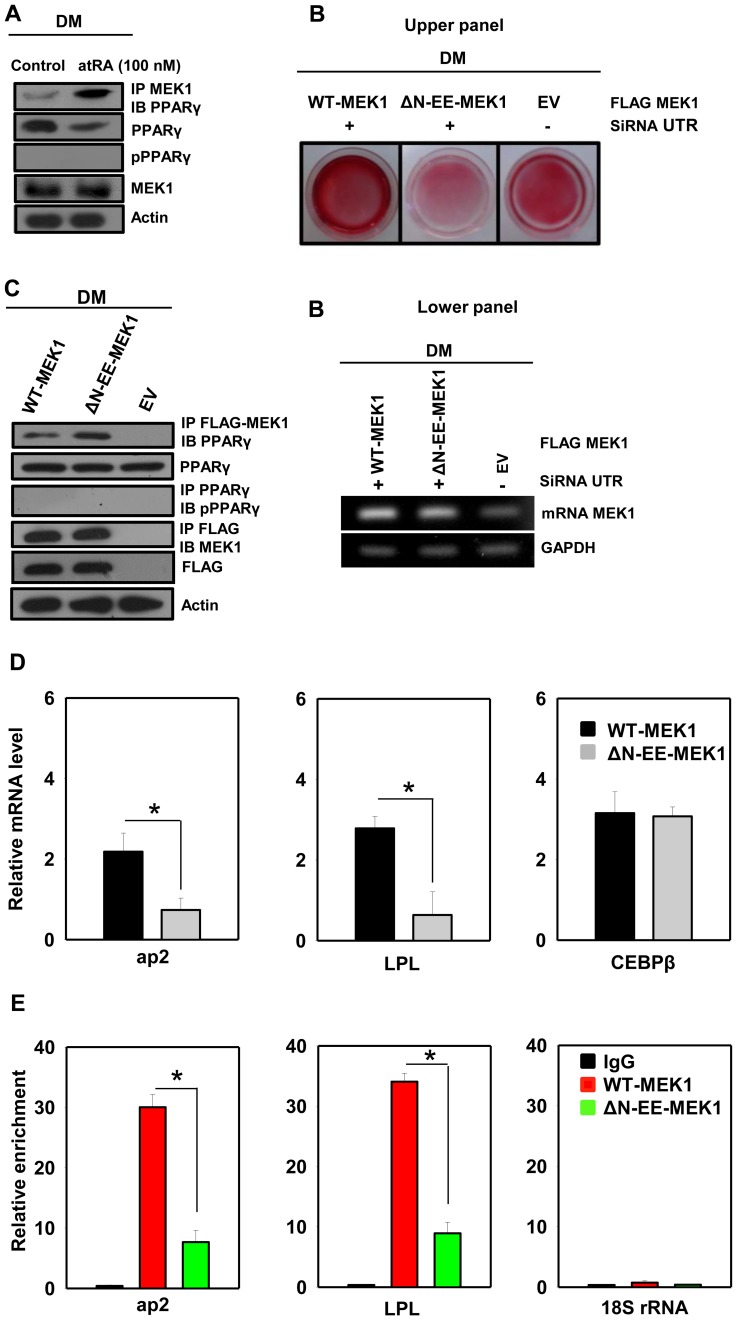
Sequestration of PPARγ by MEK1 in the nucleus of atRA treated 3T3-L1 adipocytes. PPARγ colocalization with MEK1 in control and atRA (100 nM, 48 h) and DM containing CoA treated adipocytes were monitored by (**A**) Co-immunoprecipitation in the nuclear extract. (**B**) Adipocyte differentiation as monitored by Oil Red O staining, in response to ectopic expression of FLAG tagged wild type MEK1 and export deficient constitutively active ΔN-EE-MEK1 in endogenously silenced MEK1 background. (**C**) Colocalization of FLAG-MEK1 (WT and ΔN-EE-MEK1) with endogenous PPARγ, as monitored by the co-immunoprecipiation experiment in the nuclear extract is shown in the right panel. Other immunoblots, RT-PCR constitute the inputs. (**D**) FLAG tagged wild type MEK1 and ΔN-EE-MEK1 were ectopically transfected into 3T3-L1 cell line. qPCR was performed in these ectopically expressed cells to analyse the expression of PPARγ target genes. (**E**) The ChIP assays was performed on these target genes promoters to examine the recruitment of PPARγ. The results are expressed as relative to untreated cells after normalization to 18S rRNA. Error bars show SD. *P<0.05 vs. controls. Four biological repetitions of experiments, each of which was conducted in triplicate.

MEK1 has been reported to directly interact with the master regulator of adipogenesis, PPARγ, and induces its export [42]. We wondered about the effects on the adipogenesis program that this interaction would have upon nuclear sequestration of MEK1. Upon atRA treatment, a significant nucleoplasmic colocalization was observed among PPARγ and MEK1, as evidenced by immunoprecipitation experiments with the nuclear extract (Fig. 4A, Fig. S1B). Further, in knockdown backgrounds of endogenous MEK, ectopic expression of export-deficient and constitutively active ΔN-EE-MEK1, unlike wild type (WT) MEK1, inhibited adipogenesis as monitored by Oil Red O staining and sequesteration of PPARγ (Fig. 4B and 4C, Fig. S1C and S1D). ΔN-EE-MEK1 efficiently immunoprecipitated PPARγ over WT-MEK1 (Fig. 4C), and blocked the expression of PPARγ target genes (Fig. 4D). None of these treatments induced any appreciable change in the PPARγ phosphorylation status (Fig. 4A and 4C). Sequestration of PPARγ by MEK1 seems to modulate its DNA binding ability and functionality as a transcription factor, as shown by our chromatin immunoprecipitation experiments performed on the PPARγ key regulatory target genes *aP2* and *Lpl* [63],[64] (Fig. 4E).

### N-Ethylmaleimide And Gsno Inhibited *In Vitro* Retinoylation Of Crm1

**Figure 5:**
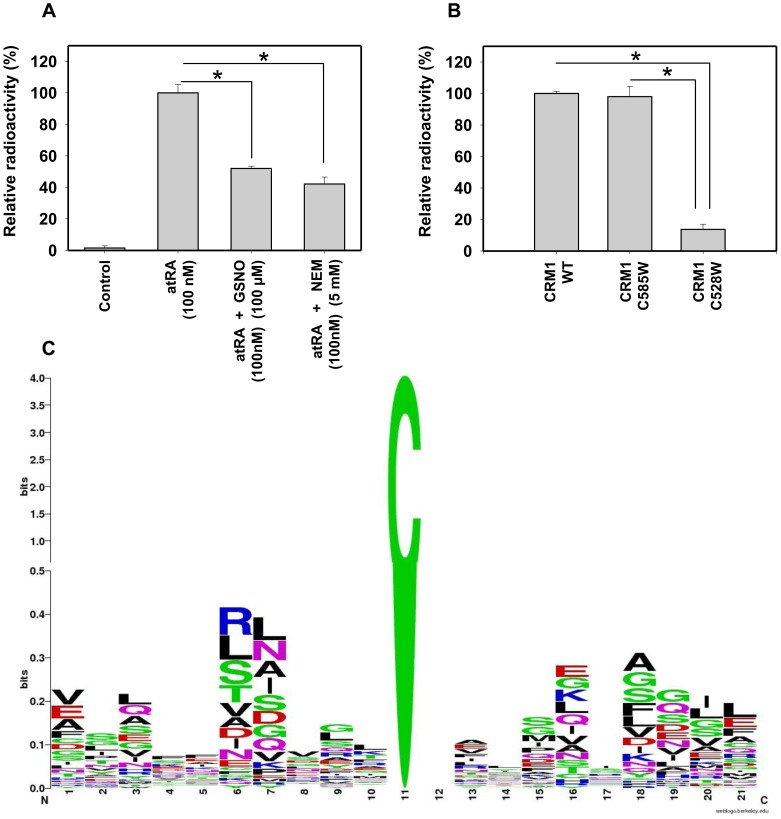
CRM1 is retinoylated. (**A**) Cell extracts of adipocytes cells, transfected with FLAG-hCRM1, were subjected to *in vitro* retinoylation in presence of N-ethylmaleimide (NEM, 5 mM) or a nitrosating agent S-nitrosoglutathione (GSNO, 100 µM). The delipidated cell extract was later pulled down for FLAG and associated radioactivity was measured by liquid scintillation. (**B**) CRM1 mutants C528W and C585W were subjected to *in vitro* retinoylation and radioactivity was measured as mentioned above. Error bars show SD. * P<0.05 vs. controls. (**C**) Analysis of sequence contexts around cysteines in known retinoylated proteins (as listed in Table 1). A composite two-scale (at Y-axis) sequence weblogo presenting a general residue preference around cysteines (central) of known retinyolated proteins. The first half of the first bit of scale on Y-axis is expanded to clearly reflect the identity of residues around the central cysteine. The web logo here is only suggestive of general preferences and may not be statistically significant for the facts that so far no retinyolated sites are known to be validated experimentally and the number of proteins taken for analysis in the study is very small.

FLAG-hCRM1 was ectopically expressed in adipocytes and the resulting cell lysate was subjected to *in vitro* retinoylation as described previously [10],[22],[23]. The extent of retinoylation was also evaluated in the presence of the alkylating agent N-ethylmaleimide (NEM, 5 mM) and the nitrosating agent S-nitrosoglutathione (GSNO, 100 µM). The cell extract was then delipidated, immunoprecipitated via FLAG and measured for associated radioactivity (Fig. 5A). While significant radioactivity was found to be incorporated in the control conditions, NEM or GSNO clearly abrogated *in vitro* retinoylation significantly. Inhibition by NEM, a sulfhydryl group reagent, confirmed the involvement of cysteine in the retinoylation reaction. Inhibition by GSNO suggests that S-nitrosylation at C528 and C585 of CRM1 [41] may interfere with retinoylation. Of the CRM1 mutants, C585W was efficiently retinoylated while C528W was not, which confirms that C528 is the residue that is involved in retinoylation (Fig. 5B). These mutants in NES-binding site of CRM1 have been reported to abolish CRM1-NES association and abrogate export of classical NES sequences fused to GFP [41]. Our study both contributes to and validates the list of proteins known to be retinoylated (Table 1). Further, we have presented data describing amino acid abundance adjacent to all cysteines of the retinoylated proteins (Fig. 5C). Interestingly at the −2 position, glycine seems to be most abundant. This corresponds with the motif identified in the case of CRM1, which interestingly also overlaps with the nitrosylation motif.

## Discussion

RA, an inducer of differentiation in several biological systems, has an inhibitory effect on adipocyte differentiation, particularly at supraphysiological concentrations (100 nm–1 µM) [22],[34],[35]. This effect does not involve RAR binding to the hormone response element RARE, and it modulates a step that is later to CEBPβ expression [7]. This study reports that atRA at a concentration of 100 nM and above, mediates retinoylation of the adipocyte proteome. This phenomenon is later to CEBPβ induction, and is largely manifested by retinoylation of CRM1. This modification disrupts the export and leads to the nuclear segregation of MEK1, which consequently sequesters PPARγ, the master regulator of adipogenesis, away from its target genes. This non-genomic pathway partially explains atRA inhibition of adipocyte differentiation.

Temporal activation of MEK1/ERK signaling at an early stage has been shown to promote adipocyte differentiation by promoting the expression of the early markers of differentiation PPARγ and CEBPα [38],[39],[65],[66]. However, a prolonged activation of the MAPK cascade could also inhibit adipocyte differentiation through phosphorylation of PPARγ, which makes it inhibitory and defunct [38],[67],[68]. MEK1 was initially thought to localize in the cell cytosol of both resting and stimulated cells [69],[70]. Research has now revealed that upon extracellular stimulation, it translocates into the nucleus [42],[52],[58],[71]. After translocation, MEK1 is rapidly exported (10-fold faster than its import) from the nucleus by its NES [69], giving the impression of cytoplasmic localization. This study provides evidence of spatial splitting of the MEK1/ERK signaling cascade by atRA. Specifically, atRA induces spatial sequestration of MEK1 in the nucleus perhaps due to retinoylation of CRM1, which as such disrupts DM-triggered temporal activation of ERK (Fig. 3). MEK1 localization to different experimental conditions was classified into five groups: (I) only cytosolic (C>>N), (II) mostly cytosolic (C>N), (III) equally distributed (C = N), (IV) mostly nuclear (C<N), and (v) only nuclear (C<<N). Maximal translocation was observed at 100 nM atRA at 24 h (60–80% nuclear). At higher concentrations of atRA the extent of translocation remained unchanged and a minuscule of MEK1 was always observed in the cytosol which does not translocate into the nucleus. Further, atRA does not prevent DM-triggered induction of CEBPβ, however, it blocks induction of its downstream target genes (PPARγ and CEBPα). This confirms that atRA blocks a step later to CEBPβ induction [7].

**Figure 6:**
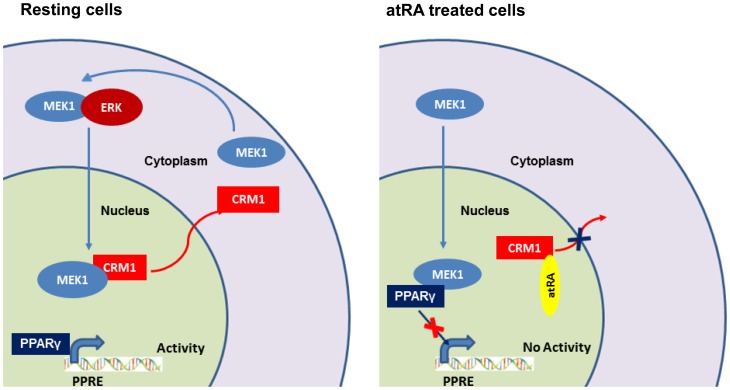
Retinoylation of CRM1 enriches MEK1 in the nucleus where it sequesters PPARγ and restricts its genomic functions.

PPARγ is the master regulator of adipogenesis and its expression and transcriptional activity at early stages is crucial for adipocyte differentiation. PPARγ regulates multiple target genes involved in differentiation and lipid metabolic pathways [72]–[76]. Interestingly, PPARγ has a CRS-like motif in its AF2 domain and has been shown to directly interact with MEK1. This induces its export upon mitogenic activation [42],[62]. These interactions lead to nuclear inactivation and cytoplasmic positioning of PPARγ. We therefore investigated the modulation of transcriptional activity of PPARγ by nuclear MEK1 (Fig. 4). Interestingly, ΔN-EE-MEK1 blocked the transcriptional activity of early PPARγ target genes, such as *Lpl* and *aP2* [63],[64]. In addition, significant nuclear colocalization of MEK1 with PPARγ was observed. ChIP experiments revealed that nuclear MEK1 reduces recruitment of PPARγ on the promoter of its target genes, which could be because of sequestration by nuclear MEK1 and explains consequent PPARγ transcriptional attenuation (Fig. 6).

This is the first study to report that atRA (100 nm–1 µM), induces retinoylation of CRM1, which can functionally abrogate the export of MEK1, an important signaling molecule. The spatial localization of MEK1 and its consequent sequestering of PPARγ though indirectly attenuate the much needed effector signaling and transcription of the adipogenesis program. Presumably, in addition to these indirect effects through CRM1, atRA might affect intricate intracellular signaling pathways directly as well. Thus, further studies are imperative to explicate the other pathways and mechanisms by which MEK1 enters the nucleus and determine whether this translocation is solely responsible for the inhibition of adipocyte differentiation. The current study identified atRA effects at early stages of adipocyte differentiation, elicited during a short-term treatment and at 100 nM concentration of atRA. It is understood that RA physiological concentrations considerably varies to specific physiological conditions and in different tissue types, and is attributable to diverse factors, such as the biosynthetic and degrading enzymes [77],[78]. It is yet to be explored, whether these pathways which are major determinant in differentiating cells mimic these effects in a prolonged RA-stimulated or in the differentiated cultures. There may be several pathways responsible for empirical effects of RA in different experimental systems, which could explain pleiotropy in retinoid action.

## Supporting Information

Figure S1 **Quantitative representation.** (**A**) pERK protein level was normalized over ERK protein level and PPARγ, CEBPα, CEBPβ were normalized over β-actin. Plots were represented as fold change by considering DM treated experimental control as 100. (**B**) Endogenous MEK1 or (**C**) ectopically expressed FLAG-MEK1 (WT or mutant) bound PPARγ protein was normalized over total PPARγ and represented in arbitrary units. (**D**) MEK1 mRNA level was analysed by semi quantitative RT-PCR and normalized over β-actin. Plot was represented by considering MEK1 expression in scrambled control as 100. Error bars show SD. *P<0.05 vs. controls. (TIF)
