# Supplementary figures and images for: Nuclear MEK1 Sequesters PPARγ and Bisects MEK1/ERK Signaling: A Non-Canonical Pathway of Retinoic Acid Inhibition of Adipocyte Differentiation
Source: PLoS One. 2014 Jun 24;9(6):e100862. doi: 10.1371/journal.pone.0100862 (PMC4069188; doi:10.1371/journal.pone.0100862)

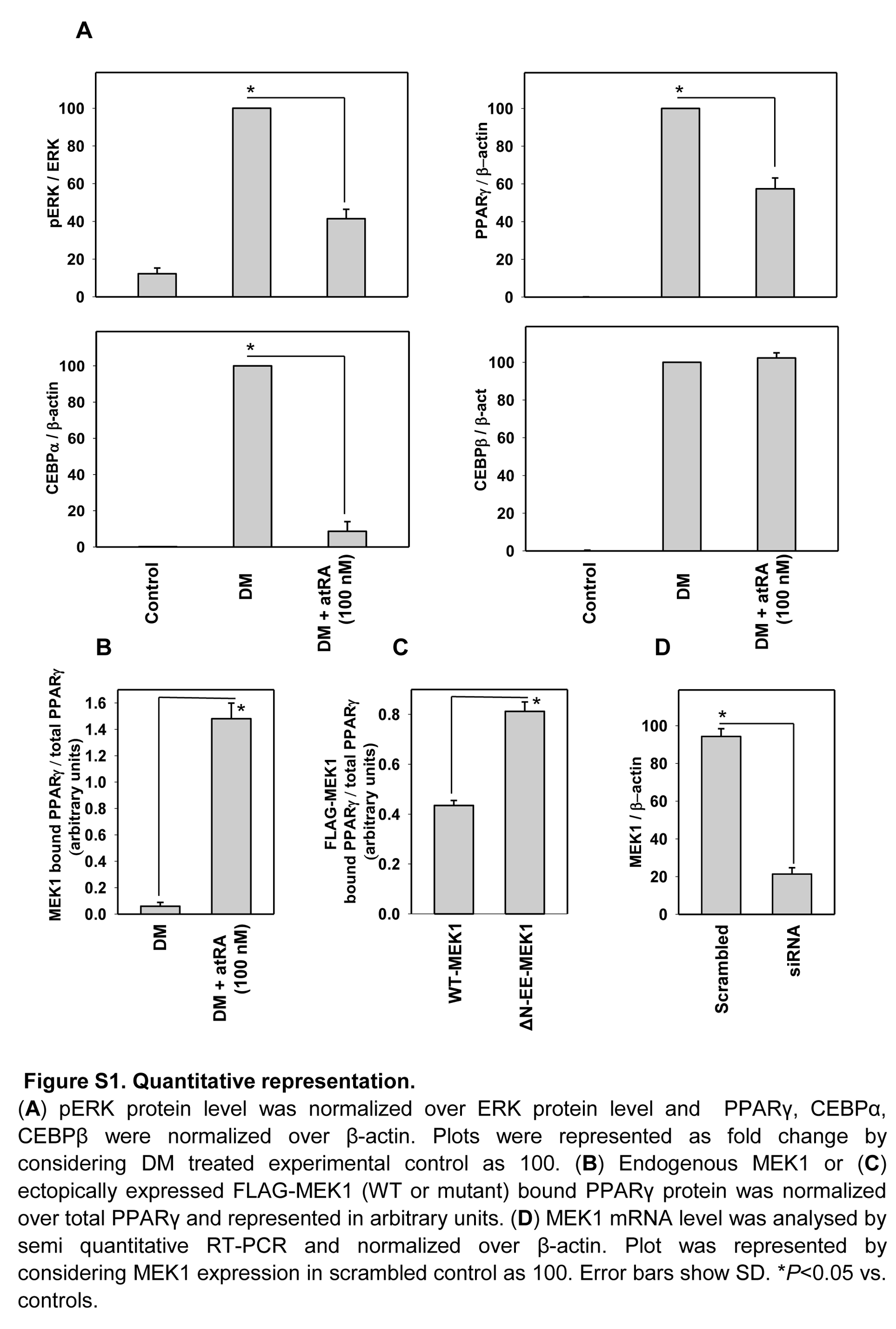

Supplement: Figure S1 — (TIF) [file pone.0100862.s001.tif]
